# Supporting Chinese American Youth Mental Health Through Culturally Responsive Parenting Workshops and Youth Social–Emotional Learning

**DOI:** 10.3390/bs16101804

**Published:** 2026-10-02

**Authors:** Cixin Wang, Elyssa Kristine Marie G. Lou, John W. Seipp, Jiayi Wang, Wenxi Yang, Jessica Momanyi

**Affiliations:** 1 Department of Counseling, Higher Education, and Special Education, College of Education, University of Maryland, College Park, MD 20742, USA; elou1@umd.edu (E.K.M.G.L.); jseipp@umd.edu (J.W.S.); yangw@umd.edu (W.Y.);; 2 Department of Psychological, Health, and Learning Sciences, College of Education, University of Houston, Houston, TX 77204, USA; jwang258@central.uh.edu

**Keywords:** social–emotional support, parent involvement, program development and evaluation

## Abstract

Chinese American youth and their immigrant parents face multiple challenges, including discrimination and acculturative stress, which negatively affect their social–emotional development and mental health. Despite elevated needs, Chinese American youth are less likely than their White peers to access school-based or community-based mental health services. We partnered with Chinese language schools to deliver two interventions: Parent–Child Connect, a culturally responsive parenting workshop, and the culturally adapted version of Merrell’s Strong Kids socio-emotional learning (SEL) curriculum to support youth mental health. A total of 49 Chinese American parents (*M* = 44.88 years, *SD* = 4.94; 90% mothers) participated in the six-week Parent–Child Connect workshop. Thirty youth (4th grade to 8th grade, 70% girls) participated in the six-week culturally adapted Strong Kids SEL program. Parents reported significant increases in positive parenting after the intervention, including warmth (*d* = 0.40), reasoning (*d* = 0.34), autonomy granting (*d* = 0.38), parent–child communication (*d* = 1.44), and perceived parenting competence (*d* = 1.11), along with a significant decrease in their own mental health difficulties/general distress (*d* = 0.33). Parents also reported a significant increase in youth prosocial behavior (*d* = 0.46). Youth reported significant increases in SEL knowledge, social–emotional competencies, and resilience (*d* = 0.99) and a marginal increase in emotion-focused coping (*d* = 0.43), after the intervention. Qualitative feedback suggested that parents and youth found the culturally tailored and interactive activities engaging, sought additional opportunities to practice coping strategies, and reported developing strategies for responding to discrimination. Findings suggest that culturally adapted parenting and SEL programs are feasible and potentially beneficial for Chinese American families when delivered in collaboration with language schools and community organizations. We discuss implications for how school psychologists, counselors, and educators can adapt interventions to better support immigrant families, promote youth social–emotional development, and improve mental health outcomes.

## 1. Introduction

Asian American youth and their immigrant parents face distinct mental health challenges that require attention. Studies show that Asian American youth report higher rates of depression compared to their White (A. C. Chen et al., 2011; J. A. Chen et al., 2019) and Latino peers (Niwa et al., 2014). Asian American youth also often encounter racial discrimination, acculturative stress, and intergenerational conflict, all of which significantly impact their mental health and well-being. These challenges may be compounded by the perpetual foreigner stereotype which casts Asian American youth as outsiders and can contribute to exclusion and victimization based on their cultural and linguistic differences (Huynh et al., 2011). Similarly, the model minority myth portrays Asian Americans as economically and educationally successful while downplaying ongoing structural barriers (Qin et al., 2008). This myth can minimize Asian American youths’ struggles, increase their risk of bullying, and worsen mental health when they face adversity (Yip et al., 2019). At the same time, intergenerational cultural and family conflict has contributed to increased mental health difficulties among young Asian Americans, including depression and suicide (Choi et al., 2020). During the COVID-19 pandemic, Asian American families, especially Chinese American families, encountered increased xenophobia amid political rhetoric and stigmatizing language such as “Chinese virus” and “Kung Flu” (Rogers et al., 2020; Stop AAPI Hate, 2022). A 2021 Pew Research Center survey found that 81% of Asian American adults reported a rise in violence against them and their community, while 32% feared that someone might threaten or physically attack them (Pew Research Center, 2021). Anti-Asian bias has continued after the official end of the pandemic (Stop AAPI Hate, 2022). For example, about 50% of Asian Americans in New York City reported experiencing hate-related incidents (e.g., insults, harassment, threats, or physical attacks) because of their race or ethnicity in 2023 (The Asian American Foundation, 2024). Among Asian American adolescents, racial discrimination during the pandemic included verbal harassment and inequitable treatment and was associated with increased risk for interpersonal violence and bullying victimization (Lu et al., 2024).

### 1.1. Barriers in Utilizing Mental Health Services in Asian American Families

Despite heightened stress and mental health needs, Asian American youth are significantly less likely to seek mental health services than their White peers (Fox & Hanes, 2023; Wang et al., 2019a). Barriers include cultural stigma surrounding mental illness, a lack of linguistically and culturally competent providers, and limited availability of services (Tiwari & Wang, 2008; Wang et al., 2019b). In Wang et al. (2019b), Asian immigrant parents identified four primary barriers to help-seeking among their adolescents: limited knowledge, negative attitudes towards help-seeking, practical access challenges, and relational barriers (e.g., lack of parent support for help-seeking). Similarly, Liu et al. (2022) explored youth perspectives on potential strategies to promote Asian American youth mental health at school. Asian American youth suggested encouraging youth to seek professional help, reducing mental health stigma, and implementing schoolwide and culturally responsive programs to promote mental health awareness (Liu et al., 2022). These findings highlight the importance of engaging Asian American parents as well as youth to promote youth social–emotional development and reduce mental health disparities within this vulnerable and underserved community.

### 1.2. Unique Challenges Faced by Asian American Youth

Because immigrant parents and their children often grow up in different national and cultural contexts, they may hold differing values, beliefs, language abilities, and communication styles (Telzer, 2010). These acculturative differences can increase family communication challenges and conflict and have been linked to poorer mental health outcomes among Asian American youth (Hwang, 2006). Moreover, parent–child conflict within Chinese American immigrant families has been associated with school misconduct and substance use (Chung et al., 2021). Additionally, Asian immigrant parents may experience minority stress, defined as chronic stress arising from racial stressors such as discrimination, internalized racism, and detachment from social support, which are linked to poorer mental health outcomes (Lei et al., 2022). Parents who experience both minority stress and acculturative stress may also adopt less positive or supportive parenting practices (Lau et al., 2011). In addition, parents may not have the tools or resources to create sufficient space for their youth to explore and cope with identity-related experiences (M. N. Li & Zhou, 2025; D. Li et al., 2025). Asian American youth also identified unique challenges they face, including perpetual foreigner stereotypes, internalized model minority myth, experience of discrimination, strict parenting practices, pressure to excel, and cultural and generational gap between parents and youth (Wang et al., 2021; Liu et al., 2022). These challenges can place significant strain on youth mental health, highlighting the need for culturally responsive interventions.

Research has consistently demonstrated that involving parents in mental health interventions improves youth outcomes, as parents are often a primary source of support when youth are coping with discrimination and other stressors (Connell & Dishion, 2008; Hanisch et al., 2010). Improving parenting practices, enhancing parent–child communication, and fostering youth SEL skills may serve as protective factors, mitigating the negative impact of these challenges on youth mental health. Given the cultural and generational gap that often exists between Asian immigrant parents and their children, it is crucial to implement parallel interventions for both parents and youth. Such interventions can help bridge cultural and generational divides by fostering mutual understanding, improving communication, and equipping families with tools to navigate cultural differences. Culturally tailored programs that address these unique needs have the potential to not only strengthen parent–child relationships but also promote resilience and mental well-being in Asian American families. Collectively, these findings highlight the importance of engaging parents, not just youth, when addressing mental health within immigrant communities.

### 1.3. Parent Involvement in Youth Social–Emotional Learning

Parents play a crucial role in the social–emotional learning (SEL) and development of their children (Sheridan & Kim, 2015). Yet, it can be challenging to engage Asian American parents in school mental health programs, due to factors such as low mental health literacy, stigma, and a lack of culturally responsive interventions. Lin and Yang (2025) introduced the cultural mismatch theory of inequality, highlighting two main barriers to Asian immigrant parents’ involvement in schools. The first barrier, an individualism–collectivism mismatch, arises when collectivistic parenting values clash with the individualistic norms prevalent in U.S. schools. The second barrier, power distance, reflects how recent immigration, relatively low socioeconomic status, and the undervaluing of Asian educational practices in Western school systems create systemic barriers to parent involvement. As a result of these barriers, as well as limited knowledge of the U.S. school system among some immigrant parents, Asian American parents often have limited participation in school-based SEL and mental health programs.

Further, existing parent training programs frequently reflect White, middle-class frameworks and values, which may not align with parenting practices and beliefs (e.g., parental authority) of many Asian immigrant families. For example, some Asian American parents may be hesitant to express affection or engage in open discussion about emotions with their children (Kim, 2007). Additionally, very few SEL programs incorporate a parent component (Cipriano et al., 2023), and when they do, minoritized parents are rarely involved, even when programs offer in-home materials or opportunities to observe SEL lessons (Drew et al., 2024). This low involvement may stem from school stakeholders’ underestimation of parents’ interest, as well as logistical and linguistic barriers (Drew et al., 2024; Ji & Koblinsky, 2009).

### 1.4. Cultural Adaptations of Social–Emotional Learning Interventions

Besides parent training, SEL interventions are a widely used approach to support youth well-being. SEL programs teach core competencies such as self-awareness, emotion regulation, relationship skills, and responsible decision-making (Collaborative for Academic, Social, and Emotional Learning, n.d.). Research repeatedly shows that SEL instruction promotes children’s social skills, self-image, academic achievement, and mental health while reducing antisocial behavior and substance use (Cipriano et al., 2023; Durlak et al., 2011). Compared with individual therapy, SEL is often more acceptable in immigrant communities because it involves learning new knowledge and skills, is delivered in a group format, and is less stigmatized (Wang et al., 2020). In addition, SEL can be embedded in educational or community settings, which are often trusted by immigrant parents (Wang et al., 2020).

Merrell’s Strong Kids curriculum is a popular SEL program with versions spanning kindergarten through high school. It has been shown to improve students’ social–emotional knowledge and coping skills while reducing internalizing symptoms (Merrell et al., 2008; Harlacher & Merrell, 2010). Each lesson takes around an hour and begins with a guided relaxation or mindfulness activity (Carrizales-Engelmann et al., 2016). Grounded in cognitive behavioral theory, the 12-lesson sequence covers key SEL topics, including understanding one’s own emotions, recognizing others’ emotions, managing anger, identifying and avoiding thinking traps, resolving interpersonal conflicts, coping with stress, fostering positive thinking, and setting personal goals. However, most studies have been conducted with European American youth.

Research on Strong Kids with minoritized youth has produced mixed findings. For example, Ryan et al. (2016) found that Strong Kids did not significantly improve social–emotional skills for Black 4th- and 5th-grade girls from low-income backgrounds, highlighting the need for cultural tailoring. In contrast, culturally adapted SEL programs for Latinx youth have shown more promise (Kurtz et al., 2023). Shi et al. (2023) adapted Strong Kids online for Latinx and Arabic-speaking English learners—reporting improvements in SEL skills and reductions in internalizing symptoms. More recently, our team adapted Strong Kids for Chinese American youth, finding improvements in youth-reported social and emotional learning knowledge and resilience, increased positive coping, and emotional regulation (Wang et al., 2026). However, SEL interventions have rarely been culturally adapted specifically for Asian American youth (A. Li et al., 2025), indicating a significant need given the growing discrimination, mental health risk, and barriers to accessing traditional mental health services. To our knowledge, our program is among the first to culturally adapt the Strong Kids program for Chinese American youth, and our program is the first to involve both parents and youth in parallel interventions of parent training and youth SEL workshops.

## 2. Current Study

While evidence-based mental health interventions for youth are available, our program is the first known SEL intervention specifically tailored to Chinese American youth (A. Li et al., 2025). Moreover, it is the only intervention we are aware of that actively involves both parents and youth, making our approach innovative and impactful. We developed a novel, culturally responsive virtual intervention involving both Chinese American parents and youth to promote positive parenting and youth SEL in order to support youth mental health (Wang et al., 2026). Specifically, our research team first developed the Parent–Child Connect intervention in 2018, which has shown promise in improving positive parenting practices, parent–child communication, sense of parenting competence, and reducing negative parenting practices (e.g., shaming, verbal hostility) (Wang et al., 2021, 2025, 2026). More recently, our research team also culturally adapted Strong Kids for Chinese American youth (Wang et al., 2026) and found it related to increased SEL knowledge and coping. Different from the previous research, we updated the intervention to better align with the post-COVID context by shifting the focus away from COVID-related discrimination and incorporating more discussions on general bullying. Additionally, we extended prior research by evaluating the effectiveness of Parent–Child Connect in reducing parental general distress and promoting youth prosocial behavior, two outcomes not previously assessed. We invited parents to attend the parenting workshop and youth to attend the youth SEL workshop.

The goal of the current study is to continue to evaluate the acceptability, social validity, and early signals of potential benefits of Parent–Child Connect and the culturally adapted Strong Kids SEL program implemented in collaboration with local Chinese language schools. Specifically, we utilized a community-based pilot multimethod intervention evaluation using a single-group pre-test–post-test design and collected both quantitative and qualitative data. We used pre- and post-tests only to quantitatively examine (a) acceptability of the Parent–Child Connect intervention; (b) early signs of the potential benefits of the Parent–Child Connect in increasing positive parenting practices, decreasing negative parenting practices, and increasing parents’ self-efficacy to support youth during discrimination; (c) early signs of the potential benefits of the culturally adapted Strong Kids in improving youth SEL knowledge, social–emotional competencies, resiliency, emotional regulation, and coping; and collected qualitative data to explore (d) participants’ perceived social validity of the program. A multimethod design was used to address these complementary but distinct research aims through multiple forms of data collection and analysis (Anguera et al., 2018). We hypothesized that (a) Parent–Child Connect intervention is feasible as indicated by strong attendance; (b) after completing Parent–Child Connect, parents would report increased positive parenting practices, perceived competence, parents’ self-efficacy to support youth during bullying, and improved youth mental health, as well as decreased negative parenting practices and general distress; (c) after completing the culturally adapted Strong Kids workshops, youth would report increased SEL knowledge, competencies, resiliency, emotional regulation, and coping; and (d) parents and youth would report positive experience with the program.

## 3. Methods

### 3.1. Participants and Procedure

#### 3.1.1. Eligibility/Recruitment

Between 2023 and 2025, we worked with Chinese language schools and community organizations to recruit Chinese American parents and youth for our interventions. Flyers were distributed through school listservs, social media platforms (e.g., WeChat), and word of mouth. Those parents interested signed up for the interventions by completing a Google sign-up form. Researchers then reviewed the sign-up form and invited eligible parents (e.g., any Chinese American parents with a school-aged youth) and youth (e.g., any Chinese American youth in 4th–8th grade) to complete a Qualtrics pre-test via email before the intervention. Upon clicking the Qualtrics link, participants were invited to provide consent and complete the survey. In order for youth to participate, parents had to provide consent, and youth had to provide assent first.

Given the community-based nature of this project, to support more families, parents were allowed to attend the workshops regardless of whether they consented to participate in the research (e.g., completing the pre- and post-surveys). For parents who were unable to attend sessions, recordings of the workshops were provided to support their learning. Recruitment and attendance data were only systematically collected for parents who provided research consent. Among consented participants, no participants formally withdrew from the study. There were no adverse events in this study.

#### 3.1.2. Participants

Among them, 49 parents provided consent, completed the pre-survey, participated in the intervention, and completed the post-intervention survey. The number of participants who completed surveys was similar across cohorts between Fall 2024 (*n* = 26) and Spring 2025 (*n* = 23). Parents ranged in age from 30 to 56 years (*M_age_* = 44.88; *SD_age_* = 4.94), were mostly mothers (*n* = 44, 90%), and held a graduate degree (Master’s/PhD; *n* = 43; 88%). All of them were born in Asia (China or Taiwan), spoke Chinese fluently, and immigrated to the U.S. as adults. Data on family income or years in the U.S. were not collected. Most parents had more than one child. Parents had children between the ages of 2 and 22, with the majority (*n* = 36; 73.47%) having at least one youth between the ages of 10 and 18. Parents were invited to rate their oldest school-aged children (ages 6 and 18) when completing the Strength and Difficulties Questionnaire (SDQ).

For parents who gave consent, we also invited their youth (4th–8th graders) to participate in a 6-week (1.5 h per week) virtual youth SEL intervention. Not all youth attended the workshops due to time conflicts. A total of 34 Chinese American youth attended the intervention and completed the pre- and post-surveys across four cohorts. Some youth attended the workshops but did not complete the surveys. Two youth were excluded from the sample due to low attendance (less than 50% of sessions), and two were dropped due to a large amount of missing data and failing an attention check in the survey, resulting in a final sample of 30 youth (30% male) across the following cohorts: Fall 2023 (*n* = 9), Spring 2024 (*n* = 5), Fall 2024 (*n* = 7), and Spring 2025 (*n* = 9). Youth ranged from 4th to 8th grade (10–14 years old). Our analysis only included parents and youth who completed both surveys.

Parents and youth samples were not matched. In Fall 2023 and Spring 2024, only youth attended the workshops. In Fall 2024 and Spring 2025, both parenting and youth SEL workshops were offered; however, not all youth attended the SEL workshops when their parents attended the parenting workshops, resulting in different parent and youth sample sizes. Participants completed the pre-intervention survey one to two weeks before the intervention and the post-intervention survey immediately after, with reminders sent to complete the survey within three weeks of finishing the intervention. Participants who completed both pre- and post-intervention surveys received e-gift cards worth $20 for parents and $10 for youth as compensation.

### 3.2. Intervention Details

All sessions were delivered by a faculty member in school psychology in addition to graduate students. The faculty member provided weekly training and supervision to graduate students (most of them were of Asian American descent) during the intervention implementation. The programs were generally delivered in the same way across cohorts following detailed PPT and teaching notes. Please see the Supplementary Materials for session details.

### 3.3. Parent Measures

#### 3.3.1. Parenting Practices

We used an abbreviated version of the Parenting Styles and Dimensions Questionnaire (PSDQ; Robinson et al., 1995), which measures parenting styles and parenting practices, such as parental warmth, reasoning, autonomy granting, and punitiveness. Parents were instructed to read each item and indicate the frequency with which they engaged in those behaviors in the past month on a 5-point Likert scale (1 = *Never* to 5 = *Always*). Sample items include “I give comfort when my child is upset” and “I punish by taking privileges away from my child with little if any explanations.” The PSDQ has sound psychometric properties (Robinson et al., 1995) and has been used with Chinese and Chinese American samples. In Chinese samples, the PSDQ has demonstrated adequate test–retest reliability (*κ* = 0.54–0.83 across subscales), interrater reliability (*κ* = 0.63–0.88 across subscales), factor loadings (0.73–0.95 across subscales), and overall adequate model fit (e.g., Fu et al., 2013). In Chinese American samples similar to the current study, the PSDQ subscales demonstrated Cronbach’s alphas between 0.59 and 0.79 (Cheah et al., 2009). In the current study, internal consistency alphas for the subscales ranged from 0.56 to 0.86 at pre-intervention and from 0.52 to 0.85 at post-intervention.

#### 3.3.2. Parenting Competency

We used the 17-item Parenting Sense of Competence scale (PSOC; Gibaud-Wallston & Wandersman, 1978). It measures a parent’s confidence in their parental role, perceived competence, capability, skill level, and problem-solving ability. Parents rated the degree to which they agreed with each statement using a 6-point scale (1 = *Strongly disagree* to 6 = *Strongly agree*). A sample item includes “Being a good parent is a reward in itself.” The tool has shown good reliability and validity (e.g., Gilmore & Cuskelly, 2009) and has been used with Asian American samples (e.g., Wang et al., 2021). Wang et al. (2021) found Cronbach’s alpha of 0.72 and 0.82 at pre- and post-intervention. In the current study, the measure also demonstrated good internal reliability, with alpha of 0.65 and 0.71 at pre- and post-intervention, respectively.

#### 3.3.3. Communication Skills

We used an adapted version of the 10-item Family Communication Scale (FCS; Olson & Barnes, 2004) to measure parental satisfaction with parent–child communication. Instead of asking about communication within the whole family, the items were reworded to specifically ask about communication between parent and youth. Participants indicated the extent to which they agreed with each item on a 6-point scale (1 = *Strongly disagree* to 6 = *Strongly agree*). A sample item includes “I can calmly discuss my ideas and beliefs with my child when we disagree.” The tool has good psychometric properties (Smith et al., 2009). In two samples of Chinese parents, the FCS demonstrated good internal consistency (α = 0.91), convergent validity via positive correlations with multiple family communication scales, and discriminant validity via stronger correlations with the Short Form-12 Health Survey Mental Component than the Physical Component (Guo et al., 2021). In the current study, the FCS showed good internal consistency at pre-intervention (*α* = 0.74) and post-intervention (*α* = 0.91).

#### 3.3.4. Confidence in Responding to a Child’s Experiences of Bullying/Discrimination

We used the Parents’ Confidence in Responding to a Child’s Experiences of Bullying Measure (Wang et al., 2025, 2026). The measure was modified based on relevant items from the Teacher Efficacy for Antibullying Scale (TEAS; Gregus et al., 2017). Instead of measuring teachers’ efficacy, the items were reworded to measure parents’ confidence in responding to their child(ren)’s experiences of bullying or discrimination. The scale consisted of five items rated on a 4-point scale (0 = *Not at all* to 3 = *A lot*). Sample items include “I can influence how my child responds to bullying/discrimination” and “I can help my child cope with bullying/discrimination.” Two previous studies with Asian American parents showed strong Cronbach’s alphas ranging from 0.70 to 0.89 (Wang et al., 2025, 2026). In the current study, the measure demonstrated good internal consistency with alphas of 0.79 and 0.91 at pre- and post-intervention, respectively.

#### 3.3.5. Parent Mental Health

We also measured parents’ mental health/general distress using the 21-item Depression, Anxiety, and Stress Scale (DASS-21; Antony et al., 1998). This measure has been used widely and has demonstrated good reliability and validity in prior research (Sinclair et al., 2012). Parents rated how much each statement applied to them on a 4-point scale (0 = *Did not apply to me at all* to 3 = *Applied to me very much or most of the time*). Sample items include “had no patience” and “can’t experience any positive feeling.” We calculated the total score for analysis. In the current study, the measure demonstrated good internal consistency (*α* = 0.93 and 0.95 at pre- and post-intervention, respectively). Subscales also indicated strong internal consistency, with values ranging from 0.83 to 0.90.

To calculate DASS-21 scores, raw subscale scores were doubled to correspond with DASS-42 score interpretation and norms. Recalculated scores were compared to the symptomology severity bands identified by Lovibond and Lovibond (1995). Based on these norms, 59.2% (*n* = 29) of parents exhibited elevated mental health symptomology at baseline. For depressive symptoms, 14.3% of parents reported mild symptoms (10–13), 14.3% moderate (14–20), and 6.1% severe or extremely severe (≥21). For anxiety, 22.5% of parents reported mild symptoms (8–9), 12.2% moderate (10–14), and 12.2% severe or extremely severe (≥15). For stress, 12.2% of parents reported mild symptoms (15–18), 10.2% moderate (19–25), and 8.2% severe or extremely severe (≥26).

#### 3.3.6. Youth Mental Health

Parents reported on youth mental health using the Strengths and Difficulties Questionnaire (SDQ; Goodman, 1997). The SDQ is a widely used measure of youth mental health and has demonstrated good reliability and validity in prior research (Du et al., 2008; Zhu et al., 2024). The measure includes five subscales: emotional difficulties, conduct difficulties, hyperactivity symptoms, peer problems, and prosocial behavior. Parents rated each item on a 3-point Likert scale (0 = *Not true* to 2 = *Certainly true*). Sample items include “Often loses temper,” “Generally well behaved, usually does what adults request,” and “Often fights with other youth or bullies them.” We calculated a total difficulties score using the first four subscales, alongside a separate prosocial behavior score. In the current study, the SDQ demonstrated good internal consistency at pre-intervention (total difficulties: α = 0.84; prosocial behavior: α = 0.77) and post-intervention (total difficulties: α = 0.90; prosocial behavior: α = 0.77). Two items with negative item-total correlations were dropped from analyses due to poor item fit: “many worries or often seems worried” and “gets along better with adults than with other youth.”

### 3.4. Youth Measures

#### 3.4.1. Social–Emotional Assets and Regulation

Youth in the Fall 2023 and Spring 2024 cohorts (*n* = 14) reported SEL skills using the 53-item Social–Emotional Assets and Regulation Scale (SEARS; Cohn et al., 2009). The SEARS measures social–emotional competencies such as friendship skills, empathy, interpersonal skills, problem-solving skills, and emotional competence. Items were rated on a 4-point Likert scale (0 = *Never* to 3 = *Always*). Sample items include “I am good at understanding the point of view of other people” and “I know how to calm down when I am stressed out or upset.” This measure demonstrated good internal consistency at pre-intervention (*α* = 0.94) and post-intervention (α = 0.97).

#### 3.4.2. Coping

Coping strategies were assessed with the 28-item Brief COPE (Carver, 1997). The Brief COPE consists of 14 subscales of two items each: active coping, use of informational support, positive reframing, planning, emotional support, venting, humor, acceptance, religion, self-blame, self-distraction, denial, substance use, and behavioral disengagement. Youth reported their use of each coping strategy on a 4-point Likert scale (1 = *I would never do this* to 4 = *I would do this a lot*). Sample items include “Get emotional support from others” and “Accept the reality of the fact that it has happened.” Because these 14 two-item subscales demonstrate limited reliability, researchers have recommended aggregating them into three broader and more reliable coping domains: problem-focused coping (active coping, planning, and using instrumental support), emotion-focused coping (positive reframing, acceptance, humor, religion, and using emotional support), and avoidant coping (self-distraction, denial, venting, substance use, behavioral disengagement, and self-blame) (Cooper et al., 2008; DeDios-Stern & Lee, 2017). The internal consistency for these three broader coping domains ranged from 0.55 to 0.84 at pre-test and 0.75 to 0.82 at post-test in this study. The internal consistency for avoidant coping at pre-test was still low, possibly because youth did not use these avoidant coping strategies consistently (e.g., none of the participants used substance as a coping strategy at either time point). Mean scores were used to calculate the composite score of each domain in this study.

#### 3.4.3. Emotion Regulation

We used the Difficulties in Emotion Regulation Scale Short Form (DERS-SF; Kaufman et al., 2016) to measure emotion regulation deficits. It is the most widely used self-report measure of emotion regulation. Items were rated on a 5-point Likert scale (1 = *Almost never* to 5 = *Almost always*), with higher scores indicating greater difficulties. A sample item is “I have no idea how I am feeling.” Internal consistency was acceptable (α = 0.88 at pre-test and 0.90 at post-test).

### 3.5. Qualitative Feedback

In addition to survey measures assessing the potential benefits of intervention, we collected qualitative data from parents and youth through open-ended questions on the post-intervention survey to gain a comprehensive understanding of their experiences with the intervention. Parent participants responded to three open-ended questions on the post-survey: “How do you like the parenting workshops?”, “How can we improve the workshops for other Asian American parents in the future?”, and “What skills do you plan to use in the next few months?” Youth also completed two open-ended feedback items on the post-intervention survey: “What do you like about this class?” and “What can we do to improve this class for future students?”

### 3.6. Data Analyses and Statistical Methods

Using R version 4.5.2 through the RStudio version 2026.01.2 interface, we conducted paired-samples *t*-tests with the t.test function in the “lsr” package to assess pre- to post-intervention changes in variables of interest for both parent and youth surveys (Navarro, 2015). We used the Benjamini–Hochberg procedure for decreasing the False Discovery Rate (FDR) to correct for multiple comparisons. Adjusted *p*-values were calculated using the p.adjust function set to “BH” in the base R package (R Core Team, 2025). Reported *p*-values in the Results section have been adjusted according to this correction procedure and tested against an alpha value of 0.05. Cohen’s *d* was used to estimate the effect size of the difference between pre-test and post-test. Following Cohen’s (1988) benchmarks, we considered 0.2 as a small effect size, 0.5 as a medium effect size, and 0.8 as a large effect size.

We analyzed the qualitative data using an inductive content analysis approach, where coding categories were derived directly from the text data (Hsieh & Shannon, 2005). To begin, two coders familiarized themselves with the data by thoroughly reviewing the qualitative responses. Each coder independently developed initial codes, which were then discussed collaboratively to create a comprehensive code list. Both coders independently applied these codes to all qualitative data, and any discrepancies were resolved through discussion. The codes and labels were iteratively refined, and similar codes were grouped into broader categories. This process continued until no new or relevant information emerged from the data.

## 4. Results

### 4.1. Participant Attendance and Engagement

Given the community-based nature of this project, we did not establish a priori feasibility criteria. Parents were permitted to attend the workshops regardless of whether they consented to participate in the research. For parents who were unable to attend sessions, recordings were provided to facilitate continued learning and participation. Among the 49 parents who provided consent, most attended at least four sessions (*n* = 41, 83.7%). Among the 30 youths, most attended at least five sessions (*n* = 28, 93.3%). All participants received session materials (PowerPoint slides, handouts) regardless of whether they attended the sessions. Parent workshop presentations were recorded, and parents who missed sessions were provided with the recordings. Youth workshops were not recorded to encourage free discussion and protect confidentiality.

### 4.2. Parent Outcomes

**Figure 1:**
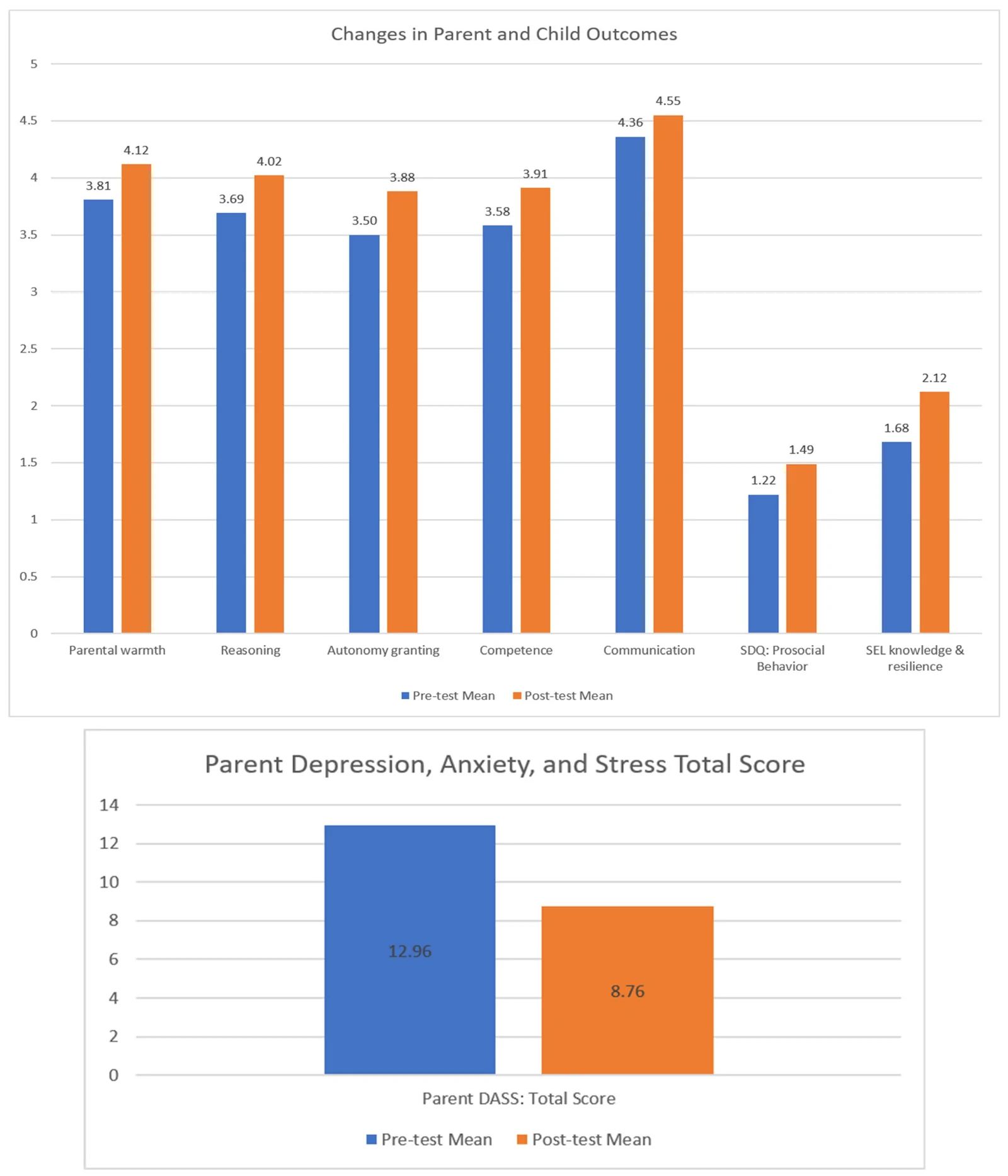
Significant Changes in Parent and Child Outcomes.

Paired-samples *t*-tests revealed significant increases in positive parenting behaviors from pre-test to post-test. Specifically, parents reported significant increases in autonomy granting (*p* = 0.027, *d* = 0.38), reasoning (*p* = 0.045, *d* = 0.34), and warmth (*p* = 0.022, *d* = 0.40). Additionally, parents reported a significant increase in parent-reported youth prosocial behavior (*p* = 0.010, *d* = 0.46). Parents reported significant increases in perceived parenting competence (*p* < 0.001, *d* = 1.11) and satisfaction in their communication with their child (*p* < 0.001, *d* = 1.44). Further, parent-reported DASS scores significantly declined from pre- to post-test (*p* = 0.04, *d* = 0.33), indicating a decrease in general distress. No significant changes were found in other parenting practices or parent-reported youth mental health difficulties (See Table 1, Figure 1).

### 4.3. Youth Outcomes

Paired-samples *t*-tests revealed a large, significant increase in SEL knowledge, competencies, and resilience (*p* = 0.013, *d* = 0.99). There was also a marginal increase in emotion-focused coping (*p* = 0.065, *d* = 0.43). There was no significant change in problem-focused coping or avoidance coping. Further, we did not observe significant changes in youth difficulties in emotion regulation (See Table 2).

### 4.4. Acceptability and Social Validity

Parents expressed positive impressions of the workshops. Many described the sessions as “very good,” “useful,” and “helpful,” with several indicating they would apply the concepts learned. A recurring theme was appreciation for the practical strategies that parents could use with their children (e.g., thinking before criticizing, I statements, active listening, and problem-solving). Participants valued the interactive nature of the sessions (e.g., “I like that parents felt free to share”; “I like the workshops a lot, especially the discussion part”). A number of parents highlighted a desire to deepen their understanding of how to effectively communicate with their children, noting the importance of emotional regulation, praise, and staying calm. Others reflected on how the strategies discussed in the workshops could positively impact youth mental health. Many appreciated the tailoring of the workshops toward Asian American parents and experiences, indicating they would recommend the training to other Asian American families.

Regarding how to improve the workshop, some parents indicated that the timing of the workshops (e.g., Saturday 7 PM) was a challenge and suggested offering an asynchronous or pre-recorded option. Others recommended tailoring content based on the ages of the children, citing differing needs across developmental stages. Additional recommendations included incorporating more relevant examples from participating parents to serve as case studies for discussion: “I wish there could be more guidance and case studies for parents in the future.” Another parent suggested collecting real-life vignettes from participants at pre-test and using these vignettes for case discussions or role-play during the future workshops.

Youth reported that they enjoyed the interactive nature of the sessions, such as the use of Kahoot and Blooket as in-session activities. Others mentioned they liked the breathing exercises, breakout rooms for discussing with peers, and the videos shown.

Regarding suggestions, youth recommended continuing the use of engaging activities (e.g., Gimkit, Blooket, Kahoot, Padlet) and YouTube videos. One youth suggested splitting discussion groups by age (younger vs. older) to allow for more age-appropriate discussions. Others suggested further exploration of stress as a topic, as well as additional discussion and strategies for youth who have experienced discrimination. Overall, youth feedback indicated the sessions were a relaxing space to learn and discuss important topics, with participants calling them “calming and fun” and noting that it “[taught] me how to decompress.”

## 5. Discussion

High rates of mental health difficulties (J. A. Chen et al., 2019) and low rates of help-seeking among Asian American youth (Fox & Hanes, 2023; Wang et al., 2019a) represent an urgent public health crisis necessitating attention and intervention. In addition, the post-pandemic social and political climate has increased the importance of Asian American families and communities accessing mental health services and highlighted the need for strengthened collaboration between families, schools, and communities. Our findings highlight a promising way to engage Chinese and Asian American parents and youth in mental health prevention programs.

Our results indicate that Parent–Child Connect is potentially beneficial in improving parenting practices and reducing parental general distress within a Chinese American sample. Parents in the study reported employing more positive practices (e.g., parental warmth, autonomy granting, and reasoning) and a higher sense of competence. This may be due to the intervention’s focus on modeling communication strategies (e.g., active listening, specific and labeled praise, emotion coaching, and problem-solving) that helped parents understand their children’s experiences and perspectives. In addition to learning parenting strategies, the group also served as a safe space and affinity space for parents to support each other by sharing personal experiences and offering emotional support to each other, which may explain why there was a significant improvement in parent mental health. Parents also reported an increase in youth prosocial behavior after the intervention. Although not statistically significant, there was a decrease in negative parenting practices (e.g., verbal hostility). Punitive parenting and shaming behaviors were low at baseline and remained low after the intervention. This finding is encouraging given the acculturation challenges and intergenerational conflicts that Asian American youth often navigate (Lau et al., 2011).

Although causal conclusions cannot be drawn, the large observed effect size for SEL knowledge, competencies, and resilience suggests promising pre–post gains among youth participants. Youth also reported learning more about coping strategies such as seeking emotional support, acceptance, and cognitive reframing to manage stress. Youth also reported an increased awareness of their emotions. In addition, youth are engaged in learning ways to respond to anti-Asian discrimination. This workshop also created a safe space or affinity space for these Chinese American youth to share relevant experiences and process their emotional responses to discrimination in a healthy way.

These improvements could be due to the consideration and discussion of culturally and contextually relevant factors (e.g., anti-Asian discrimination during COVID-19, academic pressure, culturally relevant coping strategies) and implementing culturally relevant examples, videos, activities, and mindfulness exercises during the lessons. Our findings provide preliminary support for the promise of the virtually delivered and culturally adapted Strong Kids curriculum for Asian American youth.

Both parents and youth expressed positive impressions of their respective workshops, highlighting their satisfaction with the intervention. Parents valued the provision of explicit communication strategies to use with their children, which allowed for the clear application of their acquired skills at home. Additionally, the intervention materials were tailored for Asian American parents, drawing on prior needs assessments and focus group interviews with parents and youth in the community (Liu et al., 2022; Wang et al., 2023), which may have supported the cultural relevance and acceptability of the intervention. Parents and youth also appreciated the engaging nature of the workshops. For parents, opportunities for discussion were particularly valuable, with some suggesting future workshops could include “case studies” from personal experiences to review and discuss during workshops. Youth sessions incorporated engaging activities (e.g., Kahoot; Blooket), videos, and somatic exercises. For this vulnerable age group, small-group breakout rooms led by a facilitator provided a safe and comfortable environment to discuss sensitive topics. In most lessons, an Asian American graduate student or faculty member presented and facilitated the breakout sessions. Having relevant experience and a similar identity may have also contributed to keeping youth engaged and comfortable—important components of productive youth SEL interventions.

### 5.1. Implications for Practice and Application

This study provided preliminary evidence on the potential impact of the intervention programs culturally adapted and tailored for Chinese American families: Parent–Child Connect and the culturally adapted Strong Kids program. At the same time, our findings have important implications for school psychologists, counselors, and educators who support diverse youth, especially those from immigrant families. Educators and mental health providers can work with community leaders to culturally adapt SEL interventions to support minoritized youth and involve parents in school-based mental health programs. School-based consultation (Ingraham et al., 2016; Parker et al., 2020) may also be a promising avenue for Parent–Child Connect to become embedded in schools’ practices around mental health promotion and bullying prevention. This may include a “train-the-trainer” model (Yarber et al., 2015) of working directly with school-based mental health providers and community organizations. School staff and community leaders can be trained in evidence-based and community-focused prevention programs, such as Parent–Child Connect and youth SEL, and deliver these interventions in their community to increase community buy-in of the interventions.

Additionally, the study highlights the acceptability of virtual, group-based parenting workshops. The virtual format of delivering workshops makes it easier for busy parents and youth. To increase parent engagement, researchers can solicit suggested topics and case examples from parents and youth before the workshops, and use these for discussion and role-plays. This may help by including examples relevant to the parents in attendance, building in more time for parents to engage in discussion, and better tailoring content to the age-appropriateness of the children/youth.

### 5.2. Limits of Generalizability and Future Directions

Several limitations should be noted when interpreting our findings. First, we did not include a control group in our study due to limited resources (e.g., incentives to pay participants, scheduling difficulties). Without a comparison group, it is difficult to determine the extent to which the observed improvements were directly attributable to the intervention itself versus other factors. For example, families may naturally experience improved well-being after spending six weeks in a supportive and affirming group environment with other families who share similar experiences. Some changes may reflect expectancy effects, repeated testing, or the sense of relief from validation that comes from participating in a culturally familiar setting. Future research should prioritize the inclusion of a control group to further evaluate the effectiveness of the intervention and strengthen causal interpretations. However, parent and youth feedback on perceived potential benefits from this intervention provided another indicator of the intervention effect. This study was designed as a community-based pilot multimethod intervention evaluation using a single-group pre-test–post-test design to examine acceptability and potential effects, providing initial evidence to inform future research using more rigorous designs (e.g., controlled or randomized studies). Second, the sample size is relatively small (*n* = 49 for parents and *n* = 30 for youth), as not all participants consented to participate in the research component of this project or completed the pre- and post-surveys. A very small sample (*n* = 14) of youth completed the Social–Emotional Assets and Regulation Scale (a measure of SEL knowledge, competencies, and resilience), which may limit the generalizability of the findings. The small sample size may also contribute to low internal consistency in some subscales. Future researchers can recruit more families into the intervention through wider community collaborations to further evaluate the effectiveness of the intervention.

Third, the majority of participants were highly educated mothers (88% of participants had a graduation degree), limiting the generalizability of the results to other Chinese American parents with lower education levels, limited English proficiency, and/or limited resources to attend parenting workshops. Immigrant families with lower educational levels might face more severe economic stress, limited community support, language barriers, or lower access to mental health resources. These parents may not have time to attend the live session due to work, childcare, and healthcare demands. Future studies can recruit from lower socioeconomic status communities and Title I schools. Additionally, asynchronous, prerecorded lessons or online training models may increase equitable access to the workshops, reducing the burden on families to attend, particularly for lower-income families. However, asynchronous models may limit opportunities for authentic and meaningful discussion among participants which could reduce participant engagement in the intervention. Moreover, given that study participants were mainly mothers, future studies can proactively recruit fathers and extended family members (e.g., grandparents) to increase opportunities for engagement and participation. This may include partnering with nonprofit organizations and other trusted community organizations. Furthermore, results may not be generalizable to parents from other Asian ethnic groups, given the diversity within the broader Asian American community. We need more innovative ways and resources in order to reach families from lower-income backgrounds. Instead of hosting live sessions, providing pre-recorded sessions may allow busy families to access these parenting programs. It is important to collaborate with language and cultural brokers to ensure interventions are linguistically and culturally accessible, aligning with community values. Providing funding for these collaborations and offering interventions in trusted community settings (e.g., language schools) can increase buy-in from marginalized communities. Fourth, we only collected data from parent or youth self-report. The results may be influenced by social desirability bias and/or parents’ appreciation towards the facilitator. Future studies should consider using teacher observations, behavioral outcomes, or longer-term follow-up data to more rigorously evaluate the efficacy of this intervention. Fifth, the parent and youth samples were not fully matched. Sixth, although we collected both survey and qualitative data, we did not use a mixed-methods design to fully integrate the quantitative and qualitative findings. Future studies can use a mixed-methods design to further evaluate the effectiveness of this intervention.

## 6. Conclusions

This study highlights an innovative approach to fostering youth social–emotional development and mental health through partnerships with Chinese language schools and community organizations. The findings highlight the potential benefits of culturally responsive parenting training in engaging Chinese American parents, enhancing positive parenting practices, and improving parent mental health. Additionally, the culturally adapted Strong Kids program showed potential success in promoting youth SEL knowledge, resilience, and coping skills. These results contribute to the limited body of research on culturally adapting SEL programs and parenting training for Chinese American youth and parents in educational and community settings, offering valuable insights for future interventions.

## Figures and Tables

**Table 1:** Mean, Standard Deviation, Paired *t*-test, and Cohen’s *d* for Parent Outcomes.

Outcome	*n*	Pre-Test Scores		Post-Test Scores		t-Value	Raw *p*-Value	B-H Adj. *p*-Value	Cohen’s *d*
		Mean	*SD*	Mean	*SD*				
Verbal hostility	49	2.53	0.65	2.36	0.68	−1.28	0.208	0.301	0.18
Punitive parenting	49	1.78	0.58	1.81	0.72	0.16	0.871	0.944	0.02
Parental warmth	49	3.81	0.73	4.12	0.60	2.82 *	0.007	0.022	0.40
Reasoning	49	3.69	0.79	4.02	0.67	2.39 *	0.021	0.045	0.34
Autonomy granting	49	3.50	0.80	3.88	0.73	2.67 *	0.010	0.027	0.38
Directiveness	49	2.78	0.73	2.69	0.63	−0.67	0.505	0.597	0.10
Shaming behavior	49	1.82	0.77	1.81	0.85	−0.04	0.964	0.964	0.01
Parenting competence	49	3.58	0.52	3.91	0.56	7.75 ***	5.30 × 10^−10^	3.45 × 10^−9^	1.11
Communication	49	4.36	0.55	4.55	0.74	10.07 ***	1.99 × 10^−13^	2.58 × 10^−12^	1.44
Self-efficacy for bullying intervention	49	3.12	0.57	3.30	0.63	1.47	0.148	0.240	0.21
SDQ: Total Difficulties	49	0.56	0.31	0.51	0.35	−1.01	0.318	0.414	0.14
SDQ: Prosocial Behavior	49	1.22	0.46	1.49	0.45	3.22 **	0.002	0.010	0.46
Parent DASS: Total Score	49	25.92	18.80	17.52	19.54	2.33 *	0.024	0.045	0.33

Note. Competence = Parental Sense of Competence; Communication = Parental Satisfaction with Communication; B-H adj. *p*-values have been adjusted using the Benjamini–Hochberg correction procedure for False Discovery Rate (FDR); * *p* < 0.05, ** *p* < 0.01, *** *p* < 0.001.

**Table 2:** Mean, Standard Deviation, Paired *t*-test, and Cohen’s *d* for Youth Outcomes.

Outcome	*n*	Pre-Test		Post Test		t-Values	Raw *p*-Values	B-H adj. *p*-Values	Cohen’s d
		Mean	*SD*	Mean	*SD*				
SEL knowledge & resilience	14	1.68	0.41	2.12	0.55	3.71 *	0.003	0.013	0.99
Emotion regulation difficulties	30	2.10	0.73	2.11	0.76	0.11	0.912	0.912	0.02
Coping skills:									
Problem-focused	30	2.74	0.73	2.90	0.68	1.86	0.073	0.121	0.34
Emotion-focused	30	2.43	0.52	2.70	0.57	2.35^+^	0.026	0.065 ^+^	0.43
Dysfunctional/avoidant	30	2.12	0.39	2.18	0.49	0.73	0.474	0.592	0.13

Note. B-H adj. *p*-values have been adjusted using the Benjamini–Hochberg correction procedure for False Discovery Rate (FDR); ^+^ 0.05 < *p* < 0.10, * *p* < 0.05.

## Data Availability

Data from this study are available upon reasonable request from the corresponding author.

## Supplementary Materials

The following supporting information can be downloaded at: https://www.mdpi.com/article/10.3390/bs16101804/s1, Supplemental Materials S1. Session Schedule for Parent-Child Connect workshop; Supplemental Materials S2. Schedule for Youth SEL Class (1.5 h per session. Adapted based on Merrell’s Strong Kids).
