# Supplementary material for: Supporting Chinese American Youth Mental Health Through Culturally Responsive Parenting Workshops and Youth Social–Emotional Learning
Source: Behav Sci (Basel). 2026 Oct 2;16(10):1804. doi: 10.3390/bs16101804 (PMC13701493; doi:10.3390/bs16101804)
Supplement: Supplementary file 1 [file behavsci-16-01804-s001.zip › behavsci-4403662-supplementary.pdf]

Supplemental Materials S1. Session Schedule for Parent-Child Connect workshop

Session	Session Topics	Content
1	Raising children in America	1. Understand the unique challenges faced by Asian American adolescents (e.g., perpetual foreigner stereotypes and "Model minority myth")<br>2. Understand brain development and how it impacts adolescent behavior<br>3. Broader definition of success (emotional wellbeing as an important part of success)<br>4. How to show love using different methods<br>5. Importance of self-care and stress management<br>6. Understand the strengths of Asian American families
2	Effective praise	1. How to give effective praise<br>2. Discuss common parenting practices (e.g. harsh discipline, controlling, and shaming) and when they may be problematic<br>3. Importance of balancing autonomy granting and parental control
3	Effective communication	1. Cultural differences in communication styles (direct vs indirect communication)<br>2. Empathic and non-judgmental listening<br>3. Open-ended questions<br>4. "I" statement
4	Emotion coaching	1. Be aware of child's emotions and view emotions as teachable opportunities<br>2. Help child label emotions<br>3. Communicate empathy and understanding<br>4. Teach child emotional regulation and relaxation strategies
5	Problem-solving skills	1. Problem-solving steps<br>2. Rewards and consequences/limit setting<br>3. Identify cognitive distortions and cognitive restructuring
6	Respond to bullying & school resources	1. Discuss bullying at school for Asian American students<br>2. Teach youth skills to respond to bullying/discrimination<br>3. School-based resources to support students<br>4. Review

Supplemental Materials S2. Schedule for Youth SEL Class (1.5 hours per session. Adapted based on Merrell's Strong Kids)

Session	Topics	Specific Content
1	Introduction to Social Emotional Learning (SEL) and emotional awareness	1. Learn about SEL and why it is important<br>2. Name different emotions<br>3. Identify others' emotions<br>4. Identify own character strength
2	Understanding others' emotions & empathy, effective communication	1. Identify emotional intensity<br>2. Learn about connections between thoughts and feelings<br>3. Learn how to change thoughts
3	Dealing with stress and anger, effective communication	1. Learn about the anger model<br>2. Brainstorm coping strategies<br>3. Learn communication strategies<br>4. Learn I statements to help resolve conflict
4	How to identify thinking traps	1. Learn the relationship between thoughts, emotions and behaviors<br>2. Identify different types of thinking traps/errors

5	How to avoid thinking traps and use evidence to evaluate the accuracy of thoughts and information	1. Learn strategies to avoid thinking traps<br>2. Practice (through videos & games)
6	Dealing with bullying/discrimination	1. Learn Asian American History<br>2. Discuss how to respond to bullying and discrimination

Note. Each session included a relaxation or mindfulness activity in the session.
